# Primary non-Hodgkin lymphoma of the tongue base: the clinicopathology of seven cases and evaluation of HPV and EBV status

**DOI:** 10.1186/s13000-020-00936-w

**Published:** 2020-04-01

**Authors:** Xinyu Ren, Yin Cheng, Shafei Wu, Xuan Zeng, Xiaohua Shi, Qing Ling, Zongzhu Li, Zhiyong Liang, Beverly Wang

**Affiliations:** 1 Departments of Pathology, Molecular Pathology Research Center, Peking Union Medical College Hospital, Peking Union Medical College, Chinese Academy of Medical Science, Dongdan district Shuaifuyuan 1st, 100730 Beijing, China; 2 Departments of Pathology, Beijing Children’s Hospital, Capital Medical University, National Center for Children’s Health, Beijing, 100045 China; 3 Department of Biochemistry and Molecular Medicine, The George Washington University School of Medicine and Health Sciences, Washington, DC, USA; 4 Department of Pathology and Otolaryngology, UC Irvine School of Medicine, UC Irvine Medical Center, Irvine, USA

**Keywords:** Tongue base, Non-Hodgkin’s lymphoma, Diffuse large B-cell lymphoma, Mantle cell lymphoma, Peripheral T cell lymphoma, EBV, HPV

## Abstract

**Objectives:**

Non-Hodgkin’s lymphoma (NHL) primarily derived from the base of the tongue, is rare. Human papillomavirus (HPV) and Epstein-Barr virus (EBV) are important aetiological risk factors for tumours of the head and neck. This study describes the clinicopathological features of NHL in the tongue base and the status of HPV and EBV in these cases.

**Methods:**

Seven cases were identified from the Pathological Registry Database at Peking Union Medical College Hospital (PUMCH). The study utilized immunochemistry, in situ hybridization (ISH), and gene rearrangement to confirm the disease and and performed a clinical follow up for each case.

**Results:**

All 7 lymphomas were localized at the base of the tongue. Six of the cases exhibited tongue base masses with smooth surface membranes. One case presented as multiple deep ulcers. The most common histologic subtype was diffuse large B-cell lymphoma (DLBCL), which occurred in five cases. The other two cases were mantle cell lymphoma (MCL) and peripheral T cell lymphoma, not otherwise specified (PTCL, NOS). One of the DLBCL cases was positive for HPV DNA and diffusely expressed P16 protein. During the follow up period, the MCL patient and an elderly DLBCL patient died. The remaining five patients were alive through the end of follow up.

**Conclusions:**

Most lymphomas of the tongue base manifest as an endogenous mass without membranous change. The most common subtype of NHLs of the tongue base is DLBCL, and the occurrence at this site may have a good prognosis. With proper therapy, even late stage tongue base lymphomas can be suppressed and remain in remission.

## Introduction

Lymphoma is the second most common primary malignancy occurring in the head and neck behind squamous cell carcinoma, while NHL accounts for 65–90% of all lymphomas occurring in the head and neck [1, 2] .20–30% of NHLs are derived from extra-nodal sites [3] .Nonetheless, NHL with a primary site in the oral cavity is rare, and in the tongue base, even rarer [4, 5].

Extranodal NHL is complicated; it consists of a group of tumours with different pathological, clinical and prognostic characteristics [6] .Existing series presenting extranodal NHL have mainly summarized the tumours that occur in the head and neck but are not specific to the base of the tongue. Discussions concentrating on NHL of the base of the tongue have focused on the histopathology and lack details regarding progress in the treatment response and prognosis. Understanding the biological behavior of and therapeutic options for tongue lymphoma is difficult due to the paucity of cases. Microorganisms that are regularly associated with the development of NHL include EBV, HIV,etc. [7]. HPV is considered to be associated with the occurrence of oropharyngeal squamous cell carcinoma [8], therefore, we detected the infection status of the the two viruses in lymphoma of the base of the tongue. This report adds valuable knowledge to the possible virus infection status of tongue NHL, due to its rare occurrence. In addition, an understanding of these diseases will allow the development of new targeted therapies for these aggressive lymphomas.

Here we present a literature review and case series of seven patients with NHL of the tongue base. We not only report on the general clinicopathological features, including age, gender, tumour location, histological subtypes, grading and staging, but also provide important information related to prognosis and treatment. This is the first study to report on both HPV and EBV infection status in tongue base lymphoma.

## Materials and methods

### Patients’ information

Lymphoma cases were selected from 2010 to 2017 in PUMCH, and all cases were reviewed to identify lymphomas arising from the base of the tongue instead of other primary sites. Clinicopathological information including age, gender, tumour location, histological subtype, grading, staging, survival, and response to treatment was acquired from the archives. All cases were reviewed and diagnoses were confirmed based on basic morphology, immunohistochemistry staining, and rearrangement. PET-CT/CT/MRI scans of the cancerous areas were reviewed to assess the extension of the lesions, including to the bone and thorax. Bone marrow biopsy is necessary to rule out CNS involvement.

### Immunohistochemistry

Formalin-fixed, paraffin-embedded tissue blocks of enrolled cases were used to make three-micrometer-thick sections. Immunohistochemical staining was performed using a Ventana Benchmark XT Autostainer (Ventana Medical Systems, Inc., Tucson, AZ). Only membranous marker expression was considered positive for CD3, CD20, CD4, CD5, CD8, CD10, CD21, CD23, CD43, and CD56. Either membranous or cytoplasmic expression was considered positive for CD79α, Bcl-2, and CD30. Positive nucleolus staining was used to identify Bcl-6, mum-1, CyclinD1, SOX11 and Ki-67. Cytoplasmic staining was used for ALK, TIA, AE1/AE3. P16 stains the nucleolus and cytoplasm. Positive and negative controls were included in each batch of staining. Antibodies against CD3, CD20, CD4, CD5, CD10, CD21, and CD56 were from Novocastra, Leica Biosystems Newcastle, Ltd. Antibodies of CD79α, Bcl-6, Mum-1, c-Myc, Ki-67, and AE1/AE3, ALK were from Invitrogen, USA. Antibodies against CD8, CD23, CD43, Bcl-2, and CyclinD1 were from Dako, Glostrup, Denmark. CD30 antibodies were purchased from Maixin Biotech. Co. Ltd., China. TIA, SOX10 was obtained from Beijing XiYaJinQiao Biological Technology Co. Ltd. China.

### Fluorescence in situ hybridization (FISH)

Fluorescence in situ hybridization (FISH) analysis using Break Apart FISH Probes was used to detect *BCL*2, *BCL*6 and *cMYC* gene rearrangements. The FISH probes used were 18q21 for *BCL2*, 3q27 for *BCL6*, and 8q24 for *cMYC*. Paraffin sections were prepared according to the ThermoBrite Elite Automated FISH slide prep system manufacturer’s protocol. Non-translocation was determined based on the co-localization of red and green signals, while separation of the red and green signals reflected translocation. Two pathologists interpreted the FISH results using an Olympus fluorescence microscope equipped with 100× objective lens and orange/ green/4′, 6-diamid-ino-2-phenylindole filters. Three reactive samples, either tonsils or lymph nodes, were included to establish cut-off values. Cut-off values were set as previously described [9].

### EBV in situ hybridization (ISH)

EBV ISH was performed using EBV-encoded Small RNA (EBER) probes (Bond™ ready-to-use ISH, Catalogue No: PB0589, Leica Biosystems Newcastle, Ltd.) according to the manufacturer’s protocol. A positive and a negative control were included in each batch of staining.

### HPV DNA detection by in situ hybridization

For DNA detection of high-riskin situ HPV infection, biotin-labelled HPV probe solutions (Leica, Newcastle, UK) were added to formalin-fixed, paraffin-embedded tissue sections. Samples were assayed using a BOND HPV probe set specific to HPV subtypes 16, 18, 31, 33 and 51 (Bond Ready-to-Use ISH HPV Probe, CAT # PB0829) on the Leica BOND-MAX system. The appearance of brown punctate dots in the tumour cell nucleus or cytoplasm was considered positive.

### Human papillomavirus mRNA detection by RNA in situ hybridization

For the in situ detection of high-risk HPV integration at the mRNA level, the RNAscope® FFPE 2.5 HD detection kit (Brown) (CAT #322310) was used according to the manufacturer’s instructions. Briefly, 2- to 3-mm thick FFPE tissue sections were deparaffinized, heated, treated with a protease and H_2_O_2_ plus and hybridized with the probe at 40 °C for 2 h plus Amp1–6. After washing and amplification, target RNA was stained with DAB. Nuclei were counterstained with hematoxylin. Positive staining was indicated by brown punctate dots in the cytoplasm.

### T-cell receptor and immunoglobulin gene rearrangement studies

DNA was extracted from paraffin-embedded tissue using standard DNA isolation kits (QIAGEN, 56404). For immunoglobulin gene rearrangement, we used IdentiClone IGH, IGK and IGL Gene Clonality assays with gel detection (InVivoScribe Technologies, San Diego, CA, USA). For T cell receptor rearrangement, the IdentiClone TCRB, TCRG, and TCRD Gene Clonality Assays were used with gel detection (InVivoScribe Technologies, San Diego, CA, USA). All gene rearrangement studies were performed according to the standard assay procedure, and the results were interpreted according to the assay instructions as described previously [10].

### Evaluation criteria

Briefly, the criteria and parameters for diagnosing and evaluating our cases were as follows: lymphoma classifications were based on the World Health Organization Classification of Tumors of Hematopoietic and Lymphoid Tissues (Revised Fourth Edition), and staging was based on the Ann Arbor Staging System. Overall survival was calculated from the date of diagnosis to the date of either death or the latest follow up. Four treatment response classes were defined, as follows: complete response (CR, 100% resolution); partial response (PR, 50–100% resolution); no response (< 50% resolution); and progression of disease (PD, tumour enlarged after treatment). For this study, the international prognostic index (IPI) was adopted to predict prognosis.

## Results

### Clinical characteristics of 7 patients

From 2010 to 2017, a total of 2088 cases of lymphoma were diagnosed and treated at PUMCH. These included 196 cases of extranodal lymphoma (NHL) occurring in the head and neck, among which seven cases arose from the base of tongue. Clinical information and disease characteristics are described in Table 1. Patient ages ranged from the thirties to the nineties, with an average age of 61.8 years. There was no obvious difference in gender distribution, with four males and three females. As shown in Table 1, all primary lesion locations were considered at the base of the tongue. Patients first experienced from varying degrees of throat discomfort and commit to the hospital with no B symptoms. 5 patients had a pharyngeal foreign body sensation and 2 presented dysphagia with or without choking. A mass was found through radiological and laryngoscopic examinations in six patients. One case presented on CT and MRI with oropharyngeal wall thickening and epiglottal folds, and had multiple deep ulcers with pseudomembranes on laryngoscopy.

**Table 1:** Summary of clinical characters of tongue lymphomas

Case	Age (y)	Primary Site	Tumor Size (cm)	CT/MRI Findings	Clinical Symptoms	Clinical Stage		Treatment /modality and response	IPI score	OS (months)
1	80–85	Tongue base	3.0 cm	mass	Pharyngeal foreign body sensation	IIIA		R –CHOP/PR	3	97
2	70–75	Tongue base	4.6 cm	mass	Pharyngeal foreign body sensation	IVA		R-CHOP/NR and Progression	3	63(died)
3	75–80	Tongue base	2.0 cm	mass	Pharyngeal foreign body sensation	IVA		GDP + CHOP/PR	2	95
4	40–45	Tongue base	Unknown	Thicken of oropharyngeal wall and the epiglottis folds	Dysphagia, Sore throat, choking water, multi deep ulcer with Pseudomembranous	IIA		CHOP/CR	0	85
5	90–95	Tongue base	4.3 cm	mass	Dysphagia	IA		CHOP/PR	1	3 (Died)
6	45–50	Tongue base	1.5 cm	mass	Pharyngeal foreign body sensation	IA		CHOP/CR	0	69
7	35–40	Tongue base	2.1 cm	mass	Pharyngeal foreign body sensation, Sense of suffocation	IVA		R-CHOP/PR	2	40

*R-CHOP: R-rituximab C-cyclophosphamide H-doxorubicin (hydroxydaunomycin) O-vincristine P-prednisone; CHOP: C-cyclophosphamide H-doxorubicin (hydroxydaunomycin) O-vincristine P- prednisolone; GDP:G-gemcitabine D-dexamethasone P-ciaplatin; OS: overall survival; CR: completely response; PR: partial response*

None of the seven patients presented systemic symptoms (body weight loss, fever and night sweating). Three patients were at an early stage (stage I and II) and had low IPI scores (0 or 1). Four were staged at III and IV and had higher IPI scores (2 or 3). Metastasis of the regional neck lymph nodes was noted in one case at the time of diagnoses. All patients were diagnosed by either biopsy or tumor resection.

### Pathological and immunohistochemical findings

**Fig. 1:**
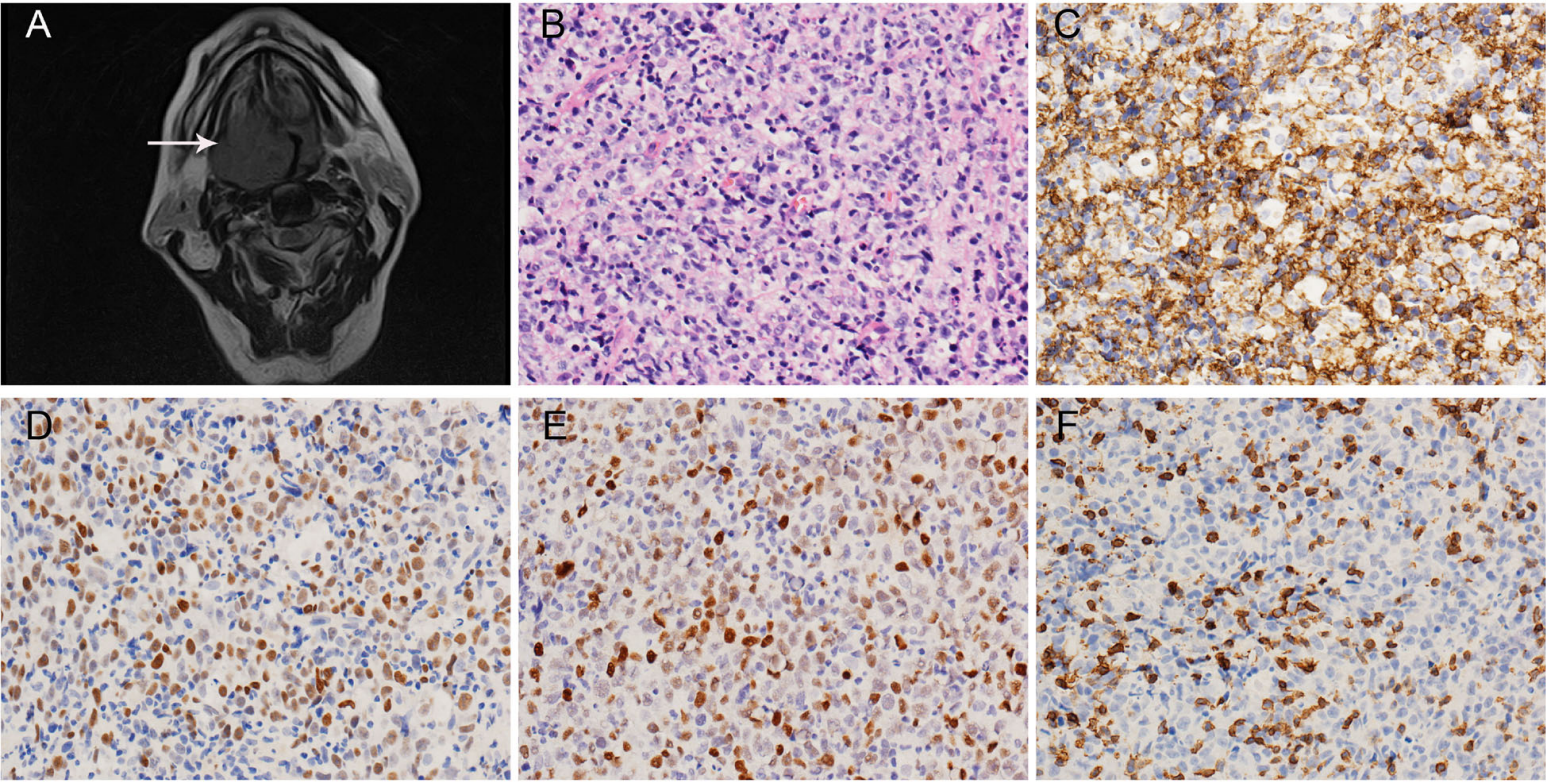
Imaging and pathological findings of DLBCL (case 5). **a**. MRI showed a mass in the base of the tongue sticking to the pharyngeal cavity and making it obviously narrow. **b**. H&E showed immunoblastic large cells with an obvious nucleolus (200 x). **c**. Tumour cells diffusely expressed CD20 (200 x). **d**. Tumour cells were positive for C-myc (200 x). **e**. Tumour cells were positive for P53 (200 x). **f**. Tumour cells were negative for CD5 (200 x)

In terms of pathological characteristics, 6 patients were diagnosed with B-cell NHL, and 1 patient was diagnosed with PTCL, NOS (Table 2). Of the 6 B-cell NHL cases, 5 were DLBCLs and 1 was MCL. Cases of PTCL and MCL are described in detail in the Results section. Of the DLBCL patients, 4 were not otherwise specified lymphomas (NOS) and 1 was T cell/histiocyte rich large B-cell lymphoma. Under the microscope, normal tissue was replaced by diffuse large atypical lymphocytes with relatively abundant cytoplasm. There were two main cytomorphological variants of the DLBCL, NOS cases: centroblastic and immunoblastic. The DLBCL, NOS cases were further divided into GC and NGC B cell like subtypes based on immunohistochemical expression of CD10, Bcl-6 and Mum1 [11]. Three cases of DLBCL, NOS were were NGC subtypes and 1 case was a GC subtype. Three out of four cases had a high Ki-67 index. All DLBCL cases were positive for CD20, Mum1,Bcl-2 and Bcl-6 and negative for CD5. One case was P53 positive (Fig. 1),and two cases expressed c-Myc(> 40%). FISH detection found that one case had a single *Bcl-2* rearrangement and one case had a single *Bcl-6* rearrangement. There were no *c-Myc* rearrangements, so there were no double or triple hit B cell lymphomas in these cases (Table 3).

**Table 2:** Summary of pathological characters of tongue lymphomas

Case	Cytological feathers	CD3	CD4	CD8	CD20	CD5	CD10	BCL-6	MUM-1	BCL2	CyclinD1	SOX11	C-MYC	P53	Ki67(%)	EBV	HPV/P16	Diagnoses
1	Large cell scattered in small lymphocyts	+			+	–	–	+	–		–	–			40	_	−/−	T cell rich large B cell lymphoma
2	Medium to large cell, partly with round nuclei and clear cytoplasm	–			+	+	+				+	+			25	_	−/−	MCL
3	moderate cells with distorted nuclear contours, infiltrate with squamous epithelium.	+	+	–	–	+					–	–			60	_	−/−	PTCL
4	Immunoblastic variant	–			+	–	–	+	+	+	–	–	–	–	80	_	−/−	DLBCL (NGC)
5	Immunoblastic variant	–			+	–	–	+	+	+	–	–	+	+	90	_	−/−	DLBCL (NGC)
6	Immunoblastic variant	–			+	–	–	+	+	+	–	–	–	–	30	_	+/+	DLBCL (NGC)
7	Centroblastic variant	–			+	–	+	+	+	+	–	–	+	–	70	–	−/−	DLBCL (GC)

*MCL: mantle cell lymphoma; PTCL: peripheral T cell lymphoma; DLBCL: diffuse large B cell lymphoma; NGC: non-germinal center type; GC: germinal center type*

**Table 3:** Literature review of peripheral T cell lymphoma

Name	year	Numberof Cases	Age/sex	Primary site of tongue	Cytologic Features	IHC characters and gene rearrangment	Clinical feature	Stage	IPI	Treatment	Survival (months)
Uherova P,et al. [12]	2002	1	56/M	–	small to intermediate, with round nuclei and abundant pale to clear cytoplasm, like marginal zone B-Cell lymphoma	CD3+,CD43+,CD45+; TCR+	–	–	–	–	–
May SA, et al. [13]	2007	1	40/F	right ventrolate	atypical small- to medium-sized lymphoid infiltrate with involvement of the overlying squamous epithelium. Having irregular nuclear contours and scant cytoplasm	CD2,3,4,5+ CD43+; TCR+ Ki-67 25%	nodule	IA	Low	CVD and CM	32 months free of disease until research published
Lee JH, et al. [14]	2014	1	59/M	right side of the tongue base	–	CD3+, TIA+, granzyme B+ CD56-Ki-67 80%	fungating mass	IIA	low	CHOP plus RT and VMAT	Died 17 months later
Narla S, et al. [15]	2016	1	50/F	left half of anterior portion	small lymphoid cells with scanty cytoplasm, irregular hyperchromatic nucleusand inconspicuous nucleoli	CD3,4,8+;TCR+Ki67 30–40%	nodule	I	low	No adjunctive therapy	1 year later lost followed up

*F: female; M: male; TCR: T cell rearrangement; CVD: cyclophosphamide, vincristine, anddexamethasone; CM: cytarabine and methotrexate; −: not mentioned;*

*CHOP: C-cyclophosphamide H-doxorubicin (hydroxydaunomycin) O-vincristine P- prednisolone; RT: radiation therapy; VMAT: volumetric modulated arc therapy*

### Viral detection

**Fig. 2:**
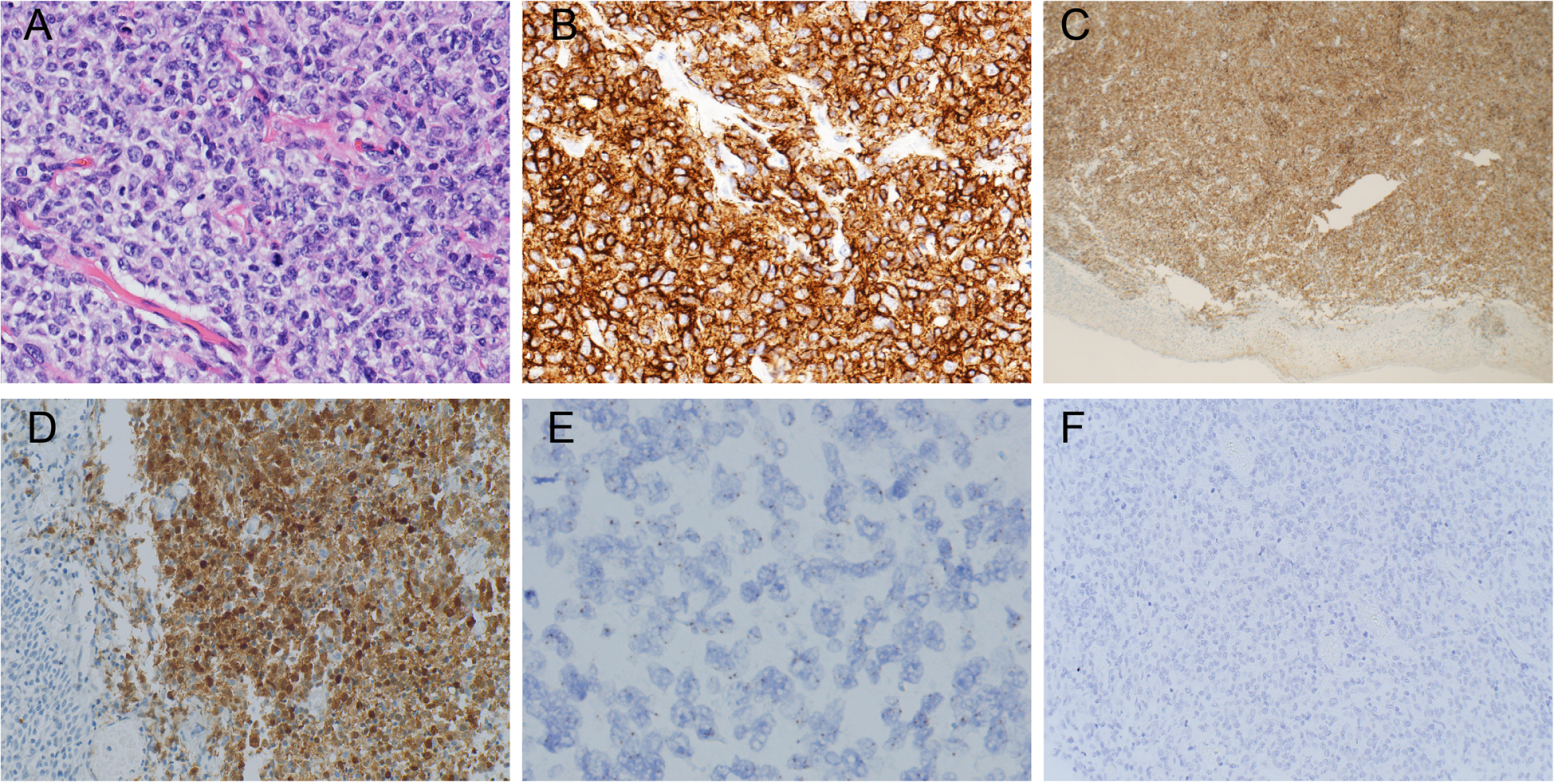
The case of DLBCL showing HPV DNA positivity (case 6). **a**. H&E showed a diffuse infiltrate of large cells with an obvious nucleolus and abundant cytoplasm (200 x). **b**. Tumour cells diffusely expressed CD20 (200 x). **c**. Immunohistochemistry staining showed diffuse and strong staining of P16 protein (40 x) **d**. Immunohistochemistry staining showed diffuse and strong staining of P16 protein (100 x). **e**. HPV DNA ISH showed brown punctate dots in the tumour cell nucleus or cytoplasm (400x).**f**. HPV RNA ISH all negative

Two probes (EBV and HPV) were used for all seven cases. All cases were negative for EBV ISH but one case was positive for HPV DNA ISH while the other six cases were negative for HPV DNA ISH. The HPV ISH positive case also had diffuse and strong expression of P16 protein as revealed by IHC, besides, HPV RNA ISH in this case is negative (Fig. 2).

As both peripheral T cell lymphoma and MCL are extremely rare in the tongue base, we would like to describe these two cases in detail as follows.

### Case of T cell lymphoma (case no. 3)

**Fig. 3:**
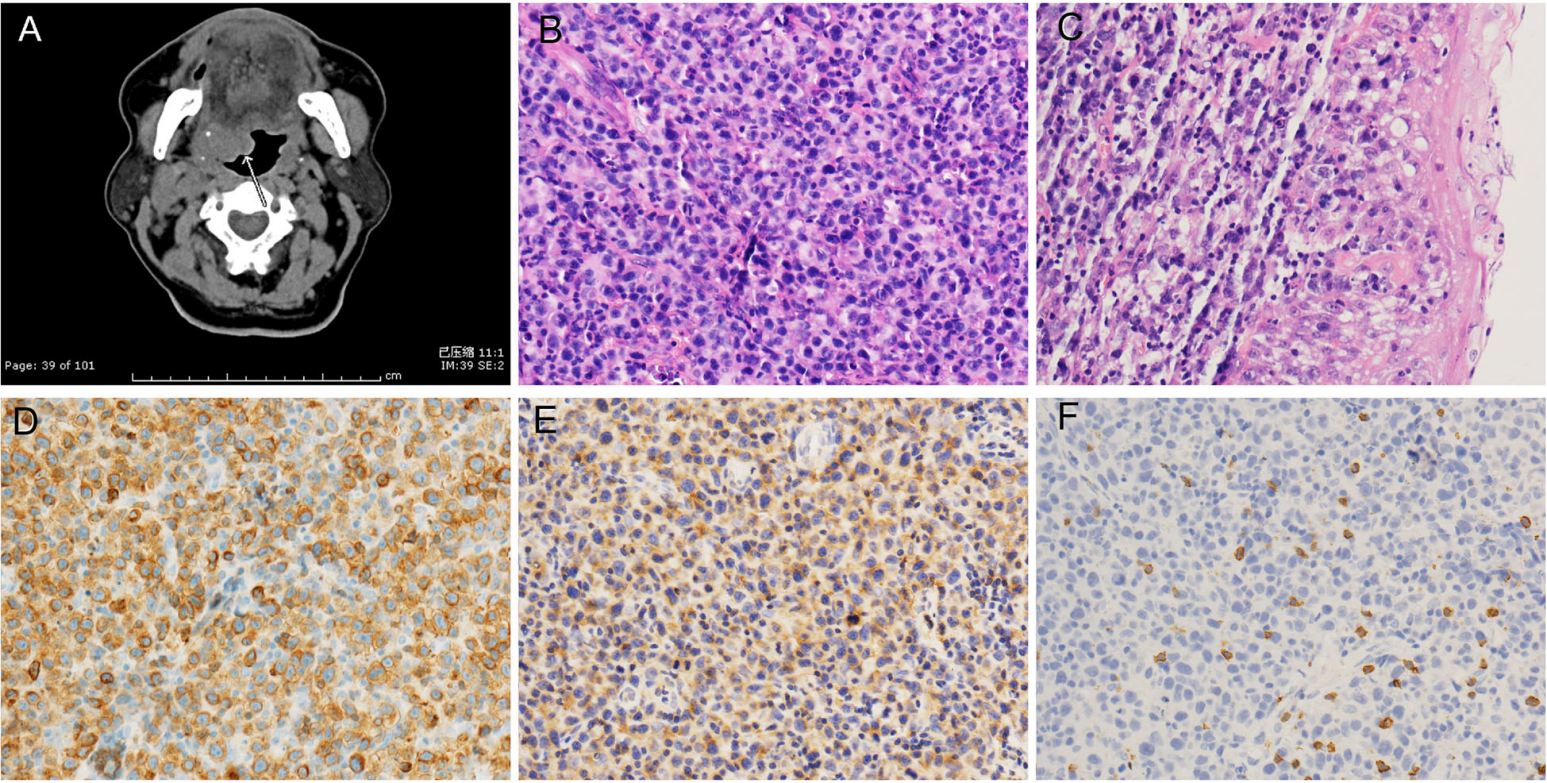
Imaging and pathological findings of PTCL (case 3). **a**. CT showed a well-bordered cystic mass. **b**. H&E showed moderate to large cells with distorted nuclear contours (200 x). **c**. Tumour cell infiltrated squamous epithelium (400x). **d**. Tumour cells diffusely expressed CD3 (200x). **e**. Tumour cells were positive for CD4 (200x). **f**. Tumour cells were negative for CD8 (200x)

A man in his fourth decade was admitted with pharyngeal foreign body sensation for two months. Imaging showed a well-bordered cystic mass (2 cm in diameter) at the right base of the tongue that extended into the pharynx, and so a biopsy was performed. In the middle power view, there were plenty of moderate to large cells with distorted nuclear contours (Fig. 3). Mitosis could be observed easily. Tumour cells expressed CD3, CD4, and CD5. In contrast, cytokeratins, CD8, CD20, CD30, ALK and CD56, TIA-1, and Granzyme B were negative. The biopsy diagnosis was peripheral T-cell lymphoma. Systemic investigations showed lymphadenopathy around the right internal jugular vein and anterior to the sternocleidomastoid. The clinical stage was IV A by the Ann Arbor staging system. His IPI score was 2(low to intermediate risk group). The patient received two cycles of GDP (gemcitabine, dexamethasone, cisplatin) and seven cycles of CHOP (cyclophosphamide, doxorubicin, vincristine, prednisone) chemotherapy. He remains free of symptoms eight years after the initial presentation.

### Case of mantle cell lymphoma (case no. 2)

**Fig. 4:**
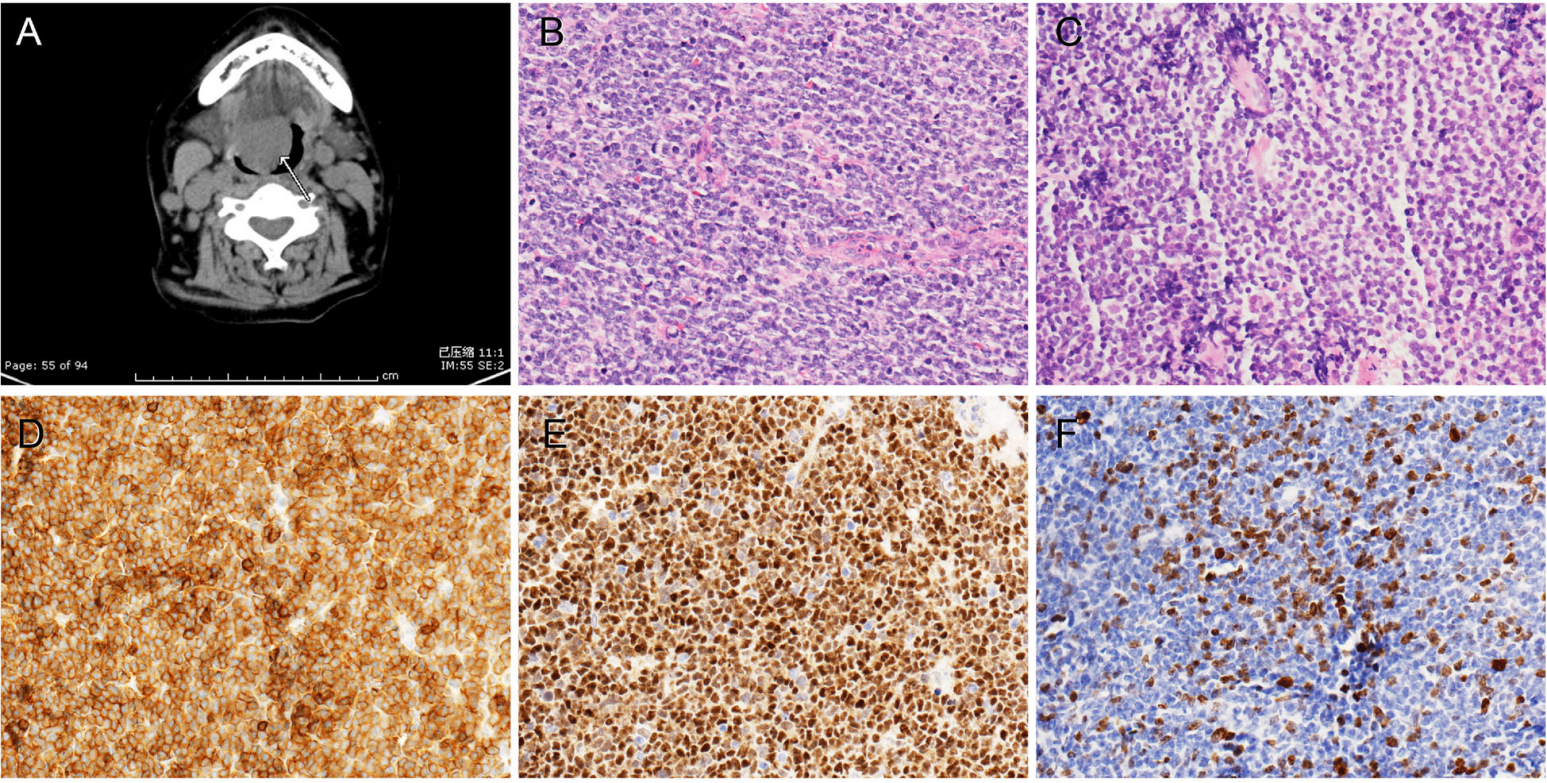
Imaging and pathological findings of MCL (case 2). **a**. CT showed an irregular soft tissue mass at the right posterior aspect of the tongue base. **b**. Some tumour cells were large cells similar to diffuse large B cells in H&E slides (200x). **c**. Some tumour cells were medium-sized with a clear cytoplasm (200x). **d**. Tumour cells were positive for CD5 (200x). **e**. Tumour cells were positive for Cyclin D1 (200x). **f**. Ki-67 staining of the tumour cells (200x)

A woman in her fourth decade was admitted with a one-month history of pharyngeal foreign body sensation. Examination and imaging (CT and MRI) showed a mass (4.6 cm × 2.8 cm × 1.5 cm) at the left base of the tongue, which was biopsied. Histologically, there was a monomorphous population of intermediate- to large-sized lymphocytes with slightly irregular indented nuclei and moderately dispersed chromatin (Fig. 4). The tumour cells were large and blastic, with a high mitotic rate, which was similar to diffuse large B lymphoma tumour cells. Immunohistochemically, the atypical lymphoid cells were positive for CD20, CD79a, PAX-5, CD5, CyclinD1 protein, and Ki-67 antigen (labelling 25%). In contrast, they did not express CD3, CD10, CD23, or TdT. The pathological diagnosis was MCL. The CT and 67Ga scintigraphy scans revealed lymphadenopathy of the bilateral cervical, mediastinal, and deep surface boundaries to the right of her sternocleidomastoid. The clinical stage was IV A. Her IPI score was 3 (high risk group). She started rituximab-CHOP(R-CHOP) regimen. Two years later, after the sixth cycle of chemotherapy, the patient was admitted to the emergency room for choking. A mass was identified in the right base of the tongue that caused breathing difficulties. Tracheotomy was performed to relieve respiratory oppression. The biopsy showed recurrence, with bone marrow involvement. Her chemotherapy regimen was changed to GDP. This patient had a partial response to chemotherapy and died 63 months after diagnosis.

### Treatment and outcome

Three patients (cases 1, 2, 7) received R-CHOP, 3 (cases 4–6) patients received CHOP, and 1 patient (case 3) received GDP and CHOP therapy. Three patients had a complete response (Table 1). The follow-up period started from the date of diagnosis until August 30, 2019, and ranged from 3 to 90 months. Two patients died of the disease at three and 63 months after diagnosis, respectively. Three patients are alive with disease and 2 are alive without disease.

## Discussion

Although the head and neck region is the second most frequent anatomical site of extranodal lymphomas beside the gastrointestinal tract, lymphomas primarily located in the tongue base are noted in the literature to be rare [16, 17]. The biological behaviours that are exclusive to the tongue base are not clear. The aetiological factors for lymphoma of the oral region other than EBV and HIV are little known.

For NHL of the head and neck, there is a logarithmic increase in incidence with increasing age [18] .The average age at disease diagnosis was 61.8 years and there were no observed gender differences. This distribution is similar to that in previous reports [18–22] .The most common location was the base of the tongue.

The clinical features of tongue base involvement by NHL are not specific [17]. Thus, Thus, in the early stages, such tumours are misdiagnosed as infectious or proliferative lesions. The most common symptoms are varying degrees of discomfort in the pharynx, such as the sensation of a foreign body or choking while drinking. Tongue musculature involvement can cause restricted movement, dysarthria, and dysphagia. Vocal cord involvement can cause choking. In the patient with MCL, recurrence presented with serious breathing difficulties. Imaging examination can help identify lesions. Except in one case, all patients exhibited a tongue base mass with smooth and intact membrane surface. Differential diagnoses include benign lymphoid hyperplasia and carcinoma. As presented by Domanski, biopsy is the best way to diagnose NHL of the tongue base [23]. In special cases, several biopsies are needed. The exceptional case here was a 45-year-old male patient with diffuse large B cell lymphoma who presented with only deep painful mouth ulcers and general symptoms, including sore throat, choking when drinking water, and difficulty swallowing. His CT and MRI scans found only thickness of the oropharyngeal wall and epiglottal folds, and a superficial biopsy revealed only inflammation. A final diagnosis was made through deep resection. The therapeutic response is related to the pathological subtype and several factors, such as old age, high grade histology, bulky lymph nodes, higher IPI score, and advanced stage [22, 24, 25].

Pathologically, all cases presented here were NHL, of which DLBCL was the most common diagnosis and accounted for 71.4% of the patients. This is consistent with head and neck research findings [6, 26]. The most common site for all cases was at the base of the tongue. Four out of five of the DLBCL cases were NOS subtypes. This is consistent with the findings from 17 DLBCL cases reported by Owosho AA et al. [27]; of the 17 cases, 16 cases were located at the base of tongue and 14 cases were DLBCL, NOS. Cases of DLBCL, NOS were further divided based on immunohistochemistry into two subtypes, GC and NGC. Among our cases, there were 1 GC and 3 NGC cases. This is slightly different from the cases reported by Owosho AA et al. [27], which comprised 9 cases of GC and 4 cases of NGC.

Although nearly 10% of DLBCL cases are reported to be EBV positive and are mainly seen in elderly people [28], EBV was not detected in any of our DLBCL cases. However, HPV infections have been identified with increasing frequency in patients with oropharyngeal squamous cell carcinoma, which is a predisposing risk factor [29]. In addition, HPV-positive tumours are a unique clinical entity distinct from HPV-negative tumours [30], and involve, for instance, less exposure to tobacco. Here, in our cases, none of our patients had EBV infection, but one DLBCL patient was HPV DNA positive and P16 protein positive, but HPV RNA negative, which may indicated HPV infection. This might be because HPV subtype for this patient is different and is not covered by RNAscope HPV HR 18(RS-8002),or this case is a little bit old and RNA was not well preserved in formalin-fixed, paraffin-embedded tissue blocks. Another reason might be HPV is not transcriptionally active in this patient; the virus integrated into the host DNA and remained inactive. The HPV subtype that often infected the cervix, were active but doesn’t do much harm to the host because the oral area was not the best breeding site for the virus. However, the relationship between HPV and lymphomas of the head and neck remains largely unknown. Viral infections, such as HIV or hepatitis C virus (HCV), can also develop in immunocompromised patients. However, HCV infection did not have a significant impact on the overall survival or event-free survival of DLBCL patients [31].HPV infection developing in this site might be due to low immunity from B cell lymphoma or HPV contributing to the development of lymphoma. Lailatul et al. showed that 74% of DLBCL cases have P16 methylation and a relatively old age [32]. Baran et al. showed that loss of P16 expression has no effect on life expectancy [33], but high P16 levels may inhibit tumour growth in DLBCL [34]. In the study of Eisuke et al., hypermethylation of the p16 promotor indicated a poor prognosis [35]. These results all indicate that HPV positivity does not have much impact on the overall survival of DLBCL patients. Our HPV-infected patient indeed had a favourable prognosis, and he was alive and free of disease when this manuscript was prepared (68 months).

Studies on the survival time for patients with DLBCL in the head and neck are controversial [24, 36, 37]; here, we added that lymphoma arising from the base of the tongue has a good prognosis. Lopez-Guillermo et al. [36] showed that patients with DLBCL located on Waldeyer’s ring (base of the tongue) often have a better prognosis than nodal DLBCL patients. Except in one case of four, all of our patients were alive through follow-up. Two patients survived more than six years. The same study also showed that lymphoma at this site is always early stage [21, 24]. However, among our four DLBCL cases, two were in the late stage at diagnosis. Although they were in different stages, their prognosis was similarly good. Most DLBCL cases of the tongue base had no Bcl-2, Bcl-6, or c-Myc rearrangement and they were sensitive to rituximab. Only one patient died of the disease. The possible reason was that the patient had several high risk factors, such as old age (in his nineties), positivity for c-Myc and P53, and co-expression of c-Myc (50%), Bcl-2 and Bcl-6 [38]. In addition, rituximab, an anti-CD20 chimeric antibody that has dramatically and favourably improved the survival rate [39], was not added to the therapeutic regimen of this case for some reason. All these factors might explain why the patient survived only 3 months after diagnosis although he was in an early stage and had a low IPI score.

PTCL, NOS occurring at the base of the tongue are rare. At the time of manuscript preparation, there were only four articles indexed in Medline that described PTCL and tongue involvement (Table 4, [12–15]). In the literature, the patients with peripheral T cell lymphoma of the tongue base were middle aged with no obvious differences in gender distribution. Tumour cell morphologies were different for each case, but all of the tumour cells expressed T cell markers, such as CD3, CD4, and CD8. Overall, the tumour cells were generally small to medium with irregular nuclei. Cytoplasmic composition also varied between cases, from abundant to scant. Patricia Uherova et al. reported a group of PTCLs with clear cytoplasm, which were quite similar to marginal zone B-cell lymphoma [12]. It is worth noting that tumour cells can infiltrate the squamous epithelium in this type of lymphoma. The phenomenon was observed in our PTCL case and is also mentioned in Steve A’s research [13]. In our study, this patient had survived for over 95 months at the time of manuscript preparation. Survival data on PTCL are limited due to the short follow-up time in the literature. One patient in the literature died 17 months after diagnosis. This may have been due to the expression of the cytotoxic marker TIA, Granzyme B, and a much higher Ki-67 index (80%), which may indicate a poor prognosis [41].

**Table 4:** Literature review of mantle cell lymphoma

Name	year	Number of Cases	Age/sex	Site of tongue	Clinical feature	Cytologic Features	Stage	Treatment	Survival
Saxman S, et al. [40]	1997	1	68/M	Right base	Mass, acute shortness of breath	–	II	CHOP and RT	Died after 18 months
Guastafierro S, et al. [17]	2008	1	62/F	right half	Mass, no symptom mentioned	small lymphocytes, with slightly irregular indented nuclei and moderately dispersed chromatin	IEA	R-CEOP Plus Rituximab maintenance therapy	Free of disease for 4 years and 5 months
Owosho, et al. [27]	2014	1	60/F	Base of tongue	–	containing many large cells similar to DLBCL	–	–	–

*F: female; M: male; CHOP: C-cyclophosphamide H-doxorubicin (hydroxydaunomycin) O-vincristine P- prednisolone; RT: radiation therapy; DLBCL: diffuse large B cell lymphoma; R-CEOP: Rituximab–Cyclophosphamide, Epirubicine, Vincristine, Prednisone; −: not mentioned*

*Cyclophosphamide, Epirubicine, Vincristine, Prednisone; −: not mentioned*

MCLs in the tongue base are even rarer. To the best of our knowledge, four cases have been reported, including our case and three cases from literature (Table 5) [17, 27, 40]. Generally, MCL patients have a median age of 60 years and a striking male predominance [42] .Three of the four cases of MCL including our case, occurred at the base of the tongue. The tumour cell composition of MCL varies greatly in the literature, from small cells with slightly irregular nuclei to large cells similar to the large cells in DLBCL, which could be misdiagnosed as DLBCL. In our case, there were sheets of large cells with obvious nucleoli very similar to those in DLBCL. MCL usually express CD5 and CyclinD1 protein. The prognosis for MCL seems to be poorer than that for DLBCL at the base of the tongue. Two patients, including our patient, died during follow-up. One patient in the literature died 18 months after diagnosis despite being in an early stage. This may be because the case occurred before drugs such as rituximab were widely available. Chemotherapy containing rituximab was considered to significantly improve survival in DLBCL and MCL patients [39, 43].

Although our case with MCL received rituximab during his second cycle of chemotherapy, he relapsed two years after the primary diagnosis. Bone marrow involvement was identified at relapse. In our case, the late stage of disease, the morphologically blastic variant [44], and involvement of neck lymph nodes were all factors that contributed to poor prognosis of this patient.

## Conclusion

In summary, NHLs in the base of the tongue are rare with nonspecific symptoms of oropharyngeal discomfort, and they could present with normal-like mucosal surfaces. Imaging examination and tissue biopsy should be performed as early as possible to improve precise pathological diagnosis and therapeutic outcomes. Tumours in this site are predominantly DLBCL subtypes in histology. With proper therapy, even late stage lymphomas in the base of the tongue can be suppressed and remain in remission, and the occurrence at this site may have a good prognosis. DLBCL with high risk factors and MCL may have unfavourable outcomes. Some cases of DLBCL may be associated with HPV infection. The number of cases in the present study was low, so further studies will be needed to better understand the relationship between HPV infection and lymphoma of the base of the tongue.

## Data Availability

The data used and/or analysed during the current study are available from the corresponding author on reasonable request.

## Abbreviations

CHOP: Cyclophosphamide, doxorubicin, vincristine, prednisone
CR: Complete response
DLBCL: Diffuse large B-cell lymphoma
EBV: Epstein-Barr virus
FISH: Fluorescence in situ hybridization
GDP: Gemcitabine, dexamethasone, cisplatin
HCV: Hepatitis C virus
HPV: Human papillomavirus
IPI: International prognostic index
ISH: In situ hybridization
NHL: Non-Hodgkin’s lymphoma
PD: Progression of disease
PR: Partial response
PTCL, NOS: Peripheral T cell lymphoma, not otherwise specified
PUMCH: Peking Union Medical College Hospital
R-CHOP: Rituximab-CHOP
